# In Vitro Activity of Two Novel Antimicrobial Compounds on MDR-Resistant Clinical Isolates

**DOI:** 10.3390/antibiotics12081265

**Published:** 2023-07-31

**Authors:** Mariam Rima, Niels Pfennigwerth, Martina Cremanns, Katarina Cirnski, Saoussen Oueslati, Sören G. Gatermann, Nicola d’Amélio, Jennifer Herrmann, Rolf Müller, Thierry Naas

**Affiliations:** 1 Team “Resist”, UMR1184 “Immunology of Viral, Auto-Immune, Hematological and Bacterial Diseases (IMVA-HB)”, INSERM, Faculty of Medicine, Université Paris-Saclay, CEA, LabEx LERMIT, 94270 Le Kremlin-Bicêtre, France; mariamrima6@gmail.com (M.R.); oueslati.saoussen@gmail.com (S.O.); 2 Department of Clinical Microbiology, Ruhr-University, 44801 Bochum, Germany; niels.pfennigwerth@rub.de (N.P.); martina.cremanns@rub.de (M.C.); soeren.gatermann@rub.de (S.G.G.); 3 Helmholtz Institute for Pharmaceutical Research Saarland (HIPS), Helmholtz Center for Infection Research and Pharmaceutical Biotechnology, Saarland University, 66123 Saarbrücken, Germany; katarina.cirnski@helmholtz-hips.de (K.C.); jennifer.herrmann@helmholtz-hips.de (J.H.); rolf.mueller@helmholtz-hips.de (R.M.); 4 German Center for Infection Research (DZIF), Partner Site Hannover, 38124 Braunschweig, Germany; 5 Bacteriology-Hygiene Unit, Assistance Publique-Hôpitaux de Paris, AP-HP Paris-Saclay, Bicêtre Hospital, 94270 Le Kremlin-Bicêtre, France; 6 Unité de Génie Enzymatique et Cellulaire UMR 7025 CNRS, Université de Picardie Jules Verne, 80039 Amiens, France; nicola.damelio@u-picardie.fr; 7 Associated French National Reference Center for Antibiotic Resistance: Carbapenemase-Producing Enterobacteriaceae, 94270 Le Kremlin-Bicêtre, France

**Keywords:** MDR, carbapenemase-producing Enterobacterales CPE, antimicrobial, in vitro susceptibility testing

## Abstract

The development of novel antibiotics is mandatory to curb the growing antibiotic resistance problem resulting in difficult-to-treat bacterial infections. Here, we have determined the spectrum of activity of cystobactamids and chelocardins, two novel and promising classes of molecules with different modes of action. A panel of 297 clinically relevant Gram-negative and Gram-positive isolates with different antibiotic susceptibility profiles, going from wild type to multi- or even extremely drug resistant (MDR, XDR) and including carbapenem-resistant isolates, were tested using broth microdilution assays to determine the minimal inhibitory concentrations (MICs), MIC50s and MIC90s of two cystobactamids derivatives (CN-861-2 and CN-DM-861) and two chelocardin derivatives (CHD and CDCHD). Cystobactamids revealed potent activities on the majority of tested Enterobacterales (MIC50s ranging from 0.25 to 4 µg/mL), except for *Klebsiella pneumoniae* isolates (MIC50s is 128 µg/mL). *Pseudomonas aeruginosa* and *Acinetobacter baumannii* showed slightly higher MIC50s (4 µg/mL and 8 µg/mL, respectively) for cystobactamids. Chelocardins inhibited the growth of Enterobacterales and *Stenotrophomas maltophilia* at low to moderate MICs (0.25–16 µg/mL) and the chemically modified CDCHD was active at lower MICs. *A. baumannii* and *P. aeruginosa* were less susceptible to these molecules with MICs ranging from 0.5 to 32 µg/mL. These molecules show also interesting in vitro efficacies on clinically relevant Gram-positive bacteria with MICs of 0.125–8 µg/mL for cystobactamids and 0.5–8 µg/mL for chelocardins. Taken together, the cystobactamid CN-DM-861 and chelocardin CDCHD showed interesting antibiotic activities on MDR or XDR bacteria, without cross-resistance to clinically relevant antibiotics such as carbapenems, fluoroquinolones, and colistin.

## 1. Introduction

Antimicrobial resistance (AMR) continues to increase, rendering the treatment of life-threatening infections with conventional antibiotics ineffective and rising the call to search for new antibiotics able to overcome the spread of this dangerous threat [1]. The rapid spread of AMR can be attributed to the unnecessary use of antibiotics, improper administration, and over-the-counter availability in some countries, along with their miss- and overuse in veterinary and agriculture fields. Additionally, the lack of novel antibiotics, relying heavily on academic research laboratories and/or small companies, due to the “big” pharmaceutical companies dropping out of antibiotic research, is potentiating the existing crisis [2,3]. However, despite all these challenges, from 2017 until 2021, more than 40 antibiotics entered clinical phase I, II, and III trials. Unfortunately, due to high attrition rates, only a minority of these will be marketed [4]. Focus of research and development of new antibiotics is now shifting towards finding molecules with (i) lower ability to develop resistance; (ii) finding novel targets to avoid cross-resistance with existing antibiotics; (iii) finding molecules with selective toxicity not to harm the host; and (iv) avoiding efflux problems especially with molecules dedicated to Gram-negative bacteria [5].

Cystobactamids and chelocardins are two novel antibiotic families that exhibit promising antibacterial activities [6,7]. Cystobactamids constitute a novel class of antibiotics putatively acting by inhibiting bacterial type II topoisomerases [8]. They are non-ribosomally synthesized molecules derived naturally from myxobacteria whose derivatives are still under investigation [9,10]. According to recent studies, CN-861-2, a synthetic cystobactamid derivative, showed very potent antibacterial activity [9,10]. Structural modification led to the synthesis of another cystobactamid derivative, CN-DM-861 [10], with improved antibacterial activity. The second family of molecules investigated in this work are chelocardins, originating from *Amycolatopsis sulphurea* [11]. Chelocardins represent atypical broad-spectrum tetracyclines and have the advantage of acting on tetracycline-resistant isolates. Their exact mode of action is not fully understood, but it is believed that they act on bacterial membranes [12]. Several derivatives have been described to overcome the natural chelocardin resistance [13]. CDCHD, an amidochelocardin, is a promising compound derived from the natural chelocardin CHD, able to act on larger number of bacterial species including *Pseudomonas* spp. [13]. This study aimed to characterize the action of two novel compounds isolated from myxobacterial and actinobacterial strains on resistant bacteria, and especially Gram-negatives (ESKAPE pathogens) resistant to carbapenems and colistin, and carbapenemase-producing Enterobacterales CPE, of which some are listed by the WHO as high priority species for which new antibiotics are urgently needed. We have investigated the antimicrobial activities of two derivatives of cystobactamids (CN-861-2 and CN-DM-861) and of chelocardins (CHD, CDCHD) against multi- and even extremely drug-resistant (MDR and XDR) clinically relevant bacterial isolates.

## 2. Results

### 2.1. Activity on Reference Strains

Minimum inhibitory concentrations (MICs) and minimum bactericidal concentrations (MBCs), determined for reference strains, can be found in Table 1. Bacteria were more susceptible to CN-DM-861 and CDCHD, compared to CN-861-2 and CHD, respectively, thus confirming the importance of chemical modifications. Low MICs for the four compounds were found for *Escherichia coli* ATCC25922, whereas high MICs were found for *Klebsiella pneumoniae* reference isolates (MICs ranging from 0.5 µg/mL to 64 µg/mL for cystobactamids and 4 µg/mL to 32 µg/mL for chelocardins). All the other bacteria had MICs ≤1 µg/mL for CN-DM-861, but higher MICs for CDCHD chelocardins (≤4 µg/mL). Based on their MICs and MBCs, cystobactamids are bactericidal, while chelocardins act more bacteriostatic for most species tested.

### 2.2. Activity on Gram-Positive Isolates

Gram-positive isolates tested are indicated in Table 2. Most of the isolates are MDR and displayed low MICs when treated with cystobactamids (ranging from 0.125 μg/mL to 8 μg/mL) or chelocardins (ranging from 0.5 μg/mL to 8 μg/mL), except for *Nocardia* and *Corynebacterium* sp., for which both cystobactamids and chelocardins display comparable MIC ranges, 8–128 μg/mL and 2–8 µg/mL (Table 2), respectively.

### 2.3. Activity on Enterobacterales

The majority of Enterobacterales strains tested were MDR, as revealed by susceptibility testing against conventional antibiotics including carbapenems (Table 3). Eighty percent of these Enterobacterales expressed a carbapenemase belonging mostly to the big 5: *Klebsiella pneumoniae* carbapenemase KPC, oxacillinase OXA-48, New Delhi metallo β-lactamase NDM, Verona integron-encoded metallo β-lactamase VIM, imipenemase IMP, but also to minor carbapenemases (German imipenemase GIM, LMB, TMB, OXA-198, etc.). These isolates were either from the French and German National Reference Centers (NRC) for Antimicrobial Resistance (AMR), the clinical bacteriology laboratory of the Bicêtre Hospital or ATCC strains (Table 1).

MIC50 for CN-861-2 and CN-DM-861 with *Klebsiella pneumoniae* isolates were very high (>128 µg/mL), while for CHD and CDCHD they were lower, 8 µg/mL and 2 µg/mL, respectively (Table 3). *Klebsiella oxytoca* (*n* = 22) and *Klebsiella variicola* (*n* = 5) were more susceptible to all compounds with MIC_50_s ≤ 1 µg/mL except for *K. variicola* MIC50 that displayed an MIC of 8 µg/mL for CN-861-2 (Table 3). MIC_50s_ for the 50 tested *E. coli* isolates were ≤2 µg/mL for the four molecules. *Salmonella* and *Shigella* displayed low MICs to cystobactamids and slightly higher values for chelocardins (Table 3). MIC_50_s of *Enterobacter and Citrobacter* species were 1 µg/mL for cystobactamids, but slightly higher for chelocardins (4 µg/mL). Variable results were found for *Serratia marcescens* and *Proteus mirabilis*. One tested *Proteus stuarti* isolate showed much higher MICs for both compounds with values of 128 μg/mL for CN-861-2, 4 μg/mL for CHD, and 8 μg/mL for CDCHD. In general, lower MIC_50_ values were found for the two chemically modified compounds CN-DM-861 and CDCHD as compared to their parental molecules.

### 2.4. Activity on Non-Fermenters

Most of the tested isolates were multidrug resistant and produced at least one ß-lactamase, including carbapenemases and are challenging in terms of antimicrobial therapy. Regarding *Pseudomonas aeruginosa*, intermediate MIC values were found for cystobactamids, with MIC_50_ of 4 μg/mL and 2 μg/mL, and MIC_90_s of 8 μg/mL for both compounds (Table 4). For chelocardins, MIC_50_s were high (>32 μg/mlL) ranging from 16 μg/mL to 256 μg/mL. For *Acinetobacter baumannii*, variable MIC values were found for cystobactamids ranging from 0.25 μg/mL to 256 μg/mL with MIC_50_ of 8 μg/mL and 4 μg/mL and MIC_90_ of 128 μg/mL and 128 μg/mL for CN-861-2 and CN-DM-861, respectively (Table 4). For chelocardins, high MIC values were found, ranging from 4 μg/mL to 64 μg/mL. Concerning *Achromobacter xylosidans,* isolates have high MICs against cystobactamids (>128 µg/mL) and slightly lower values for chelocardins (16–32 µg/mL). *Stenotrophomonas maltophilia* had variable MICs, with values of MIC_50_ of 4 μg/mL and 2 μg/mL and values of MIC_90_ of 128 μg/mL and 32 μg/mL for CN-861-2 and CN-DM-861, respectively. It also showed relatively intermediate values for chelocardins ranging from 0.5 to 32 μg/mL. *Burkholderia cepacia* showed high MIC of 256 μg/mL for cystobactamids and intermediate MICs of 8 μg/mL and 4 μg/mL for CHD and CDCHD, respectively (Table 4).

### 2.5. Colistin-Resistant Isolates

Since colistin has become one of the last options for treating carbapenem-resistant Gram-negative bacterial infections, we aimed to assess if any cross-resistance with either cystobactamids or chelocardins could be found in colistin-resistant isolates (Table 5). For *K. pneumoniae*, MICs for cystobactamids were high, irrespective of the resistance to colistin (Table 3 and Table 5), while for *E. coli*, MICs for cystobactamids remained low, irrespective of the colistin resistance mechanism.

As chelocardins are thought to also target the bacterial membrane [11], cross-resistance with colistin is a possibility. However, MICs for chelocardins remained low on colistin-resistant bacteria, thus ruling out any cross-resistance, and suggesting different mode of actions than that of colistin.

### 2.6. Fluoroquinolone-Resistant Isolates

Since cystobactamids are known to have the same target as fluoroquinolones, studying those compounds on fluoroquinolone resistant isolates allowed us to address possible cross resistance mechanism between these compounds. Seventeen fluoroquinolone-resistant Enterobacterales were tested, and the results are shown in Table 6. Except for *K. pneumoniae*, all the other tested isolates had low MICs to cystobactamids while exhibiting a high level of fluoroquinolone resistance. Our results suggest that cystobactamids may be active on fluoroquinolone resistant Enterobacterales, except for *K. pneumoniae*, which have high MIC for cystobactamids irrespective of additional resistance mechanism, suggesting a different mode of action for cystobactamids compared to fluoroquinolones.

## 3. Discussion

Despite enormous efforts in the last decade, AMR still represents one of the most challenging problems in healthcare systems worldwide. It is predicted that, unless significant measures are taken, AMR will cause 300 million deaths and a loss of 100 trillion dollars by 2050 [14]. Resistance acquisition and the mechanisms elaborated by bacteria to escape the activity of broadest antibiotics along with the absence of new antibiotics should not be ignored. Consequently, identification and characterization of novel antibiotics is of utmost importance. In this study, we aimed to characterize the action of two new classes of antibiotics from myxobacterial and actinobacterial strains regarding their potential to act on resistant bacteria, and especially Gram-negatives (ESKAPE pathogens) resistant to carbapenems and colistin, of which some are listed by the WHO as high priority species for which new antibiotics are urgently needed [15]. Especially carbapenem-resistant *A. baumannii*, carbapenem-resistant *P. aeruginosa*, and carbapenem-resistant Enterobacterales are of concern. 

Cystobactamids and chelocardins are novel antibacterial compounds that showed promising activity on subsets of bacterial isolates, and thus represent potential drug candidates [6,9,10,16,17,18]. As no clinical breakpoints are available for these compounds, the different MIC values obtained using broth microdilution were classified into three categories: species with low MICs (≤2 μg/mL); (ii) species with moderate MICs (ranging from 2 to 16 μg/mL); and (iii) species with high MICs (above 16 μg/mL) (Table 7).

Even though the mechanism of action is not completely elucidated, cystobactamids seem to act on bacterial gyrases thus inducing bacterial death by DNA synthesis inhibition [16,19,20]. Several derivatives have been chemically synthesized with improved activity, including the investigated CN-861-2 and CN-DM-861 [9], which were tested on a highly diverse panel of 297 bacterial isolates, mostly from clinical sources. The vast majority of Enterobacterales including *E. coli*, *K. oxytoca, K. variicola*, *C. freundii*, *C. braakii*, *E. cloacae*, *S. marcescens*, *Salmonella* sp., and *S. flexneri* had MICs ≤2 µg/mL (Table 3 and Table 6). *K. pneumoniae* seems to resist to the activity of cystobactamids, which could be due to the distinct capsular features of this species or due to intrinsic resistance mechanisms, as suggested for albicidin, an analogue of cystobactamids [20,21,22]. *Morganella. morganii* and *Proteus* were among the bacteria having high MICs for cystobactamids. This could be due to the specificities of the gyrases, or to outer membrane permeability problems. Nevertheless, finding derivatives of cystobactamids to bridge the species activity gap is important, as they are known to have reduced susceptibility to carbapenems and are naturally resistant to colistin. Moderate activity was shown for *P. aeruginosa* with MICs ranging from 0.5 to 32 μg/mL. *A. baumannii* seems to escape the action of cystobactamids despite the presence of a few strains having low to moderate MIC values (Table 4). Overall, the activity of cystobactamids on non-fermenting Gram-negative bacteria was not as potent. *S. maltophilia* showed diverse MIC values according to the tested strains, with MICs ranging from 0.5 to 256 μg/mL. Even though susceptibility results for clinically relevant antibiotics were available, no link between a given phenotype and higher MIC values of cystobactamids could be established. For *B. epacian*, both tested strains showed high MICs. As AMR in Gram-positive bacteria is also a growing concern, a few representatives of each species were included in the testing panel of isolates, among which *S. aureus*, *S. capitis*, and *E. feacalis* have low MICs to cystobactamids, unlike *S. pneumoniae*, *Corynebacterium* sp., *N. asteroides*, and *N. farcinica*. For all tested strains, CN-DM-861 showed better activity than CN-861-2, suggesting that it may be a promising lead that, however, still requires some optimization to include *K. pneumoniae*. As cystobactamids and fluoroquinolones have the same bacterial target, cross-resistance was something thought possible. Although many tested strains were resistant to fluoroquinolones, no obvious cross-resistance could be recorded. A recent study by Michalczyk E et al. showed that the binding of albicidin, an analog of cystobactamids, occurs at a different site then fluoroquinolones on the DNA gyrase, thus explaining the absence of cross-resistance [20]; a similar observation can be extrapolated to cystobactamids, although the exact biding site of cystobactamids to gyrase is not known. Thus, cystobactamids present an interesting alternative, especially for potential treatment of infections with fluoroquinolone-resistant bacterial isolates. 

The second family of molecules investigated in this work are chelocardins. The activity of two chelocardin derivatives, the natural CHD and the amidochelocardin CDCHD, were tested on the same selection of MDR bacterial species. For Enterobacterales, including *K. pneumoniae*, moderate MIC values were found, with a slightly improved activity for CDCHD, suggesting that the chemical modification of CHD improved its activity. With *P. aeruginosa* strains, MICs for chelocardins were high and ranged from 32 to 256 μg/mL. The activity of these molecules on *A. baumannii* was similar, which is quite disappointing since the development of compounds that could inhibit those non-fermenters has become a necessity. Gram-positive strains showed moderate MICs that ranged from 1 μg/mL to 2 μg/mL of tested bacteria. Chelocardins are atypical tetracyclines due to their similar structures. They constitute another promising class of molecules, and no cross-resistance was found with other cyclines [17]. As the exact mode of action is still not fully understood and published studies indicate they target bacterial membranes, possible cross-resistance with colistin was also investigated [11] and refuted, suggesting a different mode of action than that of colistin.

*K. pneumoniae*, *P. aeruginosa*, and *E. coli* are all Gram-negative bacteria exhibiting similar types of virulence factors [23]. They can form biofilms [24,25,26], exposing different O antigens on LPS molecules [27] and producing siderophores [28], all characteristics that have been related to the development of resistance [29]. Due to their complexity, it is hard to interpret the observed MICs considering their structural differences.

## 4. Materials and Methods

### 4.1. The Tested Compounds

The cystobactamids CN-861-2, CN-DM-861, and chelocardins CHD and CDCHD were provided by the Helmholtz Institute for Pharmaceutical Research Saarland (HIPS)—Helmholtz Center for Infection Research, Germany.

### 4.2. Bacterial Isolates

A total of 297 bacterial isolates were selected including both Gram-negative and Gram-positive isolates. These isolates corresponded to ATCC strains displaying wild-type phenotypes, but also highly drug resistant isolates that are challenging in terms of treatment options. 

Bacterial isolates were selected from the French National Reference Center for Antibiotic Resistance (F-NRC), from the bacteriology ward of the Bicêtre Hospital, France and from the German National Reference Center for Multidrug-resistant Gram-negative Bacteria, Bochum, Germany (G-NRC). There were a total of 223 Enterobacterales (*Escherichia coli* (*n* = 50), *Klebsiella pneumoniae* (*n* = 56), *Klebsiella oxytoca* (*n* = 22), *Klebsiella variicola* (*n* = 5), *Citrobacter freundii* (*n* = 20), *Citrobacter braakii* (*n* = 2), Klebsiella aerogenes (*n* = 4), *Enterobacter* sp. (*n* = 28), *Serratia marcescens* (*n* = 12), *Salmonella paratiphy* (*n* = 5), *Salmonella tiphymurium* (*n* = 2), *Salmonella* cholerasus (*n* = 3), *Salmonella* sp. (*n* = 1), *Shigella flexneri* (*n* = 1), *Morganella morganii* (*n* = 7), *Providencia stuarti* (*n* = 1), *Proteus mirabilis* (*n* = 4)); 48 non-fermentative Gram-negative isolates (*Pseudomonas aeruginosa* (*n* = 13) and *Acinetobacter baumannii* (*n* = 22); *Stenotrophomonas maltophilia* (*n* = 7), *Alcaligenes xylosidans* (*n* = 4), and *Burkholderia cepacia* (*n* = 2)); and 26 Gram-positive bacteria (*Staphylococcus capitis* (*n* = 1), *Staphylococcus aureus* (*n* = 6), *Staphylococcus epidermidis* (*n* = 3), *Streptococcus pneumoniae* (*n* = 2), *Enterococcus faecalis* (*n* = 1), *Enterococcus faecium* (*n* = 8), *Corynebacterium* sp/(*n* = 1), *Nocardia asteroides* (*n* = 3), and *Nocardia farcinica* (*n* = 1)).

### 4.3. Bacterial Identification and Susceptibility Testing of Isolates

Bacteria were identified using Matrix-Assisted Laser Desorption/Ionization Time-of-Flight (MALDI-TOF MALDI Biotyper system, Bruker Daltonics GmbH&Co. KG, Bremen, Germany). Microbroth dilution assays were used to test the selected isolates using U-bottom plates according to EUCAST MIC testing protocol and results were determined according to EUCAST guidelines, with a final bacterial concentration of 10^−5^ CFU and a total volume of 100µL in each well [30]. A wide range of concentrations was tested, going from 0.0019 µg/mL to 256 µg/mL. As no breakpoints are available for these molecules, MICs were classified into three categories: low (MICs ≤ 2 µg/mL), intermediate (4–16 µg/mL), and high (>16 µg/mL).

### 4.4. Minimum Bactericidal Activity

Minimal bactericidal concentrations MBCs were determined as recommended by EUCAST [30]. MIC plate wells with antibiotic concentrations equal to or greater than the MIC values were spread on Mueller Hinton agar to determine MBC. MBC was defined as the lowest antibiotic concentration that results in less than 0.01% surviving bacteria.

## 5. Conclusions

Our results indicate that cystobactamids and chelocardins have interesting in vitro activity on a wide range of multidrug-resistant bacteria (Gram-positive and Gram-negative) and that there is no cross-resistance with conventional antibiotics, especially with fluoroquinolones, colistin, and cyclines. Molecules derived from chemical analysis, such as CN-DM-861 and CDCHD, have improved efficacies on the tested panel of MDR pathogens.

Despite small variation among different strains, our data show that the resistance to both chelocardins and cystobactamids is highly dependent on the species, at least in the case of some of the most important ESKAPE bacteria such as *K. pneumoniae*, *P. aeruginosa*, and *E. coli*. For these bacteria, similar MIC values were found for each antibiotic class, independently of the tested isolates.

Our study on isolates resistant to antibiotics targeting the bacterial membrane (colistin) or the DNA gyrase (fluoroquinolones) was aimed at highlighting a possible cross-resistance, which would have suggested the main bacterial target of chelocardins and cystobactamids, respectively. However, even though cystobactamids have been suggested to interact with bacterial gyrase, no cross-resistance was observed with fluoroquinolones. Similarly, even though chelocardins are believed to interact with the bacterial membrane, no cross-resistance was observed with colistin. The reasons may be complex, as fluoroquinolones are structurally different from cystobactamids and may target different regions of DNA gyrase, as observed for albicidin, a related class of molecules [23]. Chelocardins are also very different from colistin and their structural similarity to tetracycline suggests that they may also target the bacterial ribosome. Structural studies and further investigations (transcriptomics and sequencing of resistant strains) will be needed to obtain deeper insight into the molecular mechanisms accounting for the activity of these interesting classes of antibacterial molecules.

## Figures and Tables

**Table 1:** MICs and MBCs for cystobactamids and chelocardins for reference strains.

	MIC (μg/mL)				MBC (μg/mL)			
Reference Strains	CN-861-2	CN-DM-861	CHD	CDCHD	CN-861-2	CN-DM-861	CHD	CDCHD
*Escherichia coli* ATCC25922	0.06	0.0019	0.5	0.25	0.125	0.015	2	2
*Klebsiella pneumoniae* ATCC15380	4	0.5	32	4	ND ^a^	ND	ND	ND
*Klebsiella pneumoniae* ATCC700603	64	32	8	4	512	256	16	16
*Enterobacter cloacae* ATCC13047	8	0.125	2	1	ND	ND	ND	ND
*Staphylococcus aureus* ATCC25923	0.25	0.25	2	4	0.5	0.25	16	8
*Staphylococcus aureus* ATCC29213	0.125	0.5	2	2	0.5	1	256	4
*Staphylococcus aureus NCTC12493*	≤0.06	≤0.06	1	1	0.06	0.125	8	2
*Enterococcus faecalis* ATCC29212	0.25	0.25	2	1	1	1	512	128
*Pseudomonas aeruginosa* ATCC27853	0.5	0.25	8	4	1	2	512	32
*Acinetobacter baumannii* ATCC17978	2	1	8	2	ND	ND	ND	ND

^a^ ND: not determined.

**Table 2:** MICs of tested Gram-positive isolates.

Species		MICs (µg/mL)			
	# of Tested Isolates	CN-861	CN-DM-861	CHD	CDCHD
*Streptococcus pneumoniae*	2	0.25–2	0.5	0.5–4	2–4
*Enterococcus faecalis* ATCC29212	1	0.25	0.25	1	2
*Enterococcus faecium* vanA	4	1–4	1–8	4–8	2–4
*Enterococcus faecium* vanB	2	0.5	0.5–2	4–8	2–4
*Enterococcus faecium Tet(M)*	2	≤0.125–0.5	≤0.125–0.25	4–8	2–8
*Staphylococcus capitis*	1	0.25	0.125	2	1
*Staphylococcus aureus* ATCC25923	1	0.25	0.25	1	4
*Staphylococcus aureus* ATCC29213	1	0.25	0.5	2	2
*Staphylococcus aureus* NCTC12493	1	0.5	≤0.125	1	1
*Staphylococcus aureus mecA*	2	≤0.125	≤0.125	4	4
*Staphylococcus aureus mecC*	1	≤0.125	0.5	4	4
*Staphylococcus epidermidis mecA*	3	≤0.125	≤0.125	2–4	2–4
*Corynebacterium* sp.	1	8	8	1	0.5
*Nocardia asteroides*	3	64–>128	>128	4–8	2–4
*Nocardia farcinica*	1	>128	>128	8	4

**Table 3:** MIC50 and MIC90 of cystobactamids (CN-861-2, CN-DM-861) and chelocardins (CDCHD and CHD) in Enterobacterales.

Species		MIC Range [MIC50] (µg/mL)				MIC90 (µg/mL)				Percentage of Resistant Isolates (%)													
	n	CN-861-2	CN-DM-861	CHD	CDCHD	CN-861-2	CN-DM-861	CDCHD	CHD	AMX	AMC	PRL	IMP	MER	ERT	CAZ	CTX	FEP	FF	CIP	LEV	TGC	CT
*E. coli* ^a^	50	0.06–256 [0.25]	0.0019–256 [0.25]	0.25–32 [2]	0.25–16 [1]	2	2	16	8	100	100	100	68	81	87	100	96	100	25	ND	81	12.5	43
*K. pneumoniae* ^b^	56	0.5–256 [128]	0.25–256 [128]	0.5–32 [8]	0.5–32 [2]	256	256	16	32	100	100	100	33	90	100	100	100	95	90	ND	90	81	46
*K. oxytoca* ^c^	22	0.25–4 [0.25]	0.25–2 [0.25]	0.25–32 [0.5]	0.25–32 [1]	2	2	8	16	100	28	100	0	ND	0	28	30	23	ND ^n^	7	7	ND	ND
*K. variicola* ^d^	5	0.5–64 [8]	0.25 [0.25]	0.5–2 [1]	1–2 [1]	16	0.25	2	2	100	0	100	0	ND	0	0	0	0	ND	0	0	ND	ND
*Enterobacter* sp. ^e^	28	0.25–64 [1]	0.25–2 [0.25]	1–16 [4]	0.5–32 [4]	64	2	16	16	100	100	100	72	72	90	100	100	90	72	ND	63	9	45
*C. freundii* ^f^	20	0.25–1 [0.25]	0.25–1 [0.25]	1–16 [4]	0.5–4 [1]	1	1	4	8	100	100	100	87	87	100	100	100	100	6	ND	93	12.5	62.5
*M. morganii* ^g^	7	2–64 [4]	0.5–1 [1]	4–16 [4]	2–32 [8]	64	1	32	16	100	100	100	100	100	100	100	100	100	100	ND	25	25	100
*Salmonella* sp. ^h^	11	0.25–5 [0.5]	0.25–5 [0.25]	0.5–4 [2]	0.25–2 [1]	0.25	0.25	2	4	ND	40	0	0	0	0	ND	0	0	40	20	ND	ND	ND
*S. marcescens* ^i^	12	1–128 [16]	0.25–32 [0.25]	0.25–4 [2]	0.5–32 [2]	128	32	32	4	100	100	100	80	80	100	100	100	100	40	ND	100	0	80
*S. flexneri* ^j^	1 ^O^	0.5	0.5	2	2					0	0	0	0	0	0	0	0	0	0	0	0	ND	ND
*K. aerogenes* ^k^	4 ^O^	0.5–8	ND	2–16	8–16					ND	ND	ND	ND	ND	ND	ND	ND	ND	ND	ND	ND	ND	ND
*C. brakii* ^k^	2	0.25–4	0.25–2	4–16	2					ND	ND	ND	ND	ND	ND	ND	ND	ND	ND	ND	ND	ND	ND
*P. stuartii ^l^*	1	128	128	4	8					ND	ND	ND	ND	ND	ND	ND	ND	ND	ND	ND	ND	ND	ND
*P. mirabilis* ^m^	4	8–32	8–32	1–4	4–16					100	100	100	100	0	100	100	100	100	0	0	100	0	100

Number of isolates with susceptibility testing results for conventional antibiotics: ^a^, 16; ^b^, 23; ^c^, 14; ^d^, 5; ^e^, 11; ^f^, 16; ^g^, 4; ^h^, 10; ^i,^ 5; ^j^, 1; ^k^, 0; ^l^, 1; ^m^, 4. ^n^, ND: no data; ^O^, for species with less than five tested isolates, MIC_50_ and MIC_90_ were not calculated; rather, MIC or a range of MIC values are presented. Antibiotic abbreviations: AMX: amoxycillin; AMC: co-amoxiclav; PRL: piperacillin; IMP: imipenem; MER: meropenem; ERT: ertapenem; CAZ: ceftazidime; CTX: cefotaxime; FEP: cefepime; FF: Fosfomycin; CIP: ciprofloxacin; LEV: levofloxacin; TGC: tigecycline; CT: colistin. ND: not determined.

**Table 4:** MIC_50_ and MIC_90_ of cystobactamids CN-861-2, CN-DM-861, and chelocardins CDCHD, and CHD on non-fermenters.

Species		MIC Range [MIC50] (µg/mL)				MIC90 (µg/mL)			
	*n*	CN-861-2	CN-DM-861	CHD	CDCHD	CN-861-2	CN-DM-861	CHD	CDCHD
*P. aeruginosa*	13 ^a^	0.5–256 [4]	0.5–32 [2]	16–256 [64]	32–256 [32]	8	8	256	256
*A. baumannii*	22 ^a^	0.5–256 [8]	0.25–256 [4]	16–64 [8]	2–64 [2]	128	128	32	64
*S. maltophilia*	7 ^a^	0.5–256 [4]	0.5–64 [2]	0.5–32 [1]	0.5–4 [1]	128	32	16	4
*A. xylosidans*	4 ^b^	128	128	16–32	4–16				
*B. cepacia*	2 ^b^	256	128–256	8	4–8				

^a^ *n*: number of isolates tested; ^b^ for species with less than five tested isolates, MIC50 and MIC90 were not calculated; rather, a range of MIC values are presented.

**Table 5:** MICs for cystobactamids and chelocardins against colistin-resistant *K. pneumoniae* and *E. coli* strains.

Species	# of Isolates	Resistance Mechanism	MIC Range [MIC50] (µg/mL)				
			CN-861-2	CN-DM-861	CHD	CDCHD	Colistin
*K. pneumoniae*	5	*mgrB* mutation	256	256	0.5–8 [4]	4–32 [16]	8–128 [64]
*E. coli*	8	Plasmid-mediated resistance *(mcr-1)*	0.25–16 [0.25]	0.25–4 [0.25]	0.25–4 [0.5]	0.25–8 [8]	4–8 [4]
*E. coli*	11	Chromosomal resistance to colistin ^a^	0.25–32 [0.25]	0.25–2 [0.25]	0.5–4 [1]	0.25–16 [0.5]	4–16 [4]

^a^ Mutations included frameshifts, IS*Kpn7* insertions, and complete Mgrb deletion.

**Table 6:** MICs of fluoroquinolone-resistant Enterobacterales tested for cystobactamids and chelocardins.

Species	Isolate #	ß-lactam Resistance	MIC Range [MIC50] (µg/mL)					
			CN-861-2	CN-DM-861	CHD	CDCHD	CIP ^a^	LEV ^b^
*K. pneumoniae*	4	2 NDM, 1 CTX-M-15, 1 OXA-48	16–>128 [>128]	2–>128 [>128]	4–32 [32]	2–8 [2]	>4	>4
*K. oxytoca*	1	OXA-48 LIKE	<0.25	<0.25	8	2	>4	>4
*E. coli*	8	6 NDM, 2 CTX-M-15	<0.25	<0.25	1–8 [2]	0.5–2 [0.5]	>4	>4
*E. cloacae*	3	1 Hyper-AmpC, 1 VIM, and 1 OXA-48	<0.25	<0.25	1–16 [1]	0.5 [1]	>4	>4
*C. freundii*	2	OXA-48 LIKE	<0.25–0.5	<0.25–0.5	8–16	1	>4	>4
*C. brakii*	1	OXA-48 LIKE	<0.25	<0.25	4	2	>4	>4

^a^ ciprofloxacin; ^b^ levofloxacin.

**Table 7:** Activity spectrum of cystobactamids (CN-861-2 and CN-DM-861) and chelocardins (CDCHD and CHD) on Gram-negative bacteria.

MICs	Cystobactamids		Chelocardins	
	CN-861-2	CN-DM-861	CDCHD	CHD
MIC ≤ 2 µg/mL	*K. oxytoca* *E. coli* *C. freundii* *E. cloacae* *Salmonella* *Shigella*	*K. oxytoca* *K. variicola* *E. coli* *C. freundii* *E. cloacae* *Salmonella* *Shigella* *M. morganii*	*K. oxytoca* *K. variicola* *E. coli* *C. freundii* *E. cloacae* *Salmonella* *Shigella* *S. maltophilia*	*K. oxytoca* *E. coli* *Salmonella* *Shigella* *S. maltophilia* *P. mirabilis* *P. stuarti* *N. asteroides*
2 µg/mL < MIC ≤ 16 µg/mL	*K. variicola* *M. morganii* *P. mirabilis* *P. aeruginosa* *A. baumannii* *S. maltophilia*	*A. baumannii*	*K. pneumoniae* *M. morganii* *P. mirabilis* *P. stuarti* *A. baumannii* *N. asteroides* *N. farcinica* *B. cepacia*	*K. pneumoniae* *M. morganii* *P. stuarti* *C. freundii* *E. cloacae* *A. baumannii* *N. farcinica* *B. cepacia*
MIC > 16 µg/mL	*K. pneumoniae* *P. stuarti* *B. cepacia* *N. asteroides* *N. farcinica*	*K. pneumoniae* *N. asteroides* *N. farcinica*	*P. aeruginosa*	*P. aeruginosa*

## Data Availability

Raw data are available upon request.

## References

[1] Ventola C.L. (2015). The antibiotic resistance crisis: Part 1: Causes and threats. Pharm. Ther..

[2] Michael C.A., Dominey-Howes D., Labbate M. (2014). The Antimicrobial Resistance Crisis: Causes, Consequences, and Management. Front. Public Health.

[3] Osman M., Al Mir H., Rafei R., Dabboussi F., Madec J.Y., Haenni M., Hamze M. (2019). Epidemiology of antimicrobial resistance in Lebanese extra-hospital settings: An overview. J. Glob. Antimicrob. Resist..

[4] Boyd N.K., Teng C., Frei C.R. (2021). Brief Overview of Approaches and Challenges in New Antibiotic Development: A Focus On Drug Repurposing. Front. Cell. Infect. Microbiol..

[5] Silver L.L. (2011). Challenges of antibacterial discovery. Clin. Microbiol. Rev..

[6] Baumann S., Herrmann J., Raju R., Steinmetz H., Mohr K.I., Hüttel S., Harmrolfs K., Stadler M., Müller R. (2014). Cystobactamids: Myxobacterial Topoisomerase Inhibitors Exhibiting Potent Antibacterial Activity. Angew. Chem. Int. Ed..

[7] Mitscher L.A., Juvarkar J.V., Rosenbrook W., Andres W.W., Schenck J.R., Egan R.S. (1970). Structure of chelocardin, a novel tetracycline antibiotic. J. Am. Chem. Soc..

[8] Groß S., Schnell B., Haack P.A., Auerbach D., Müller R. (2021). In vivo and in vitro reconstitution of unique key steps in cystobactamid antibiotic biosynthesis. Nat. Commun..

[9] Hüttel S., Testolin G., Herrmann J., Planke T., Gille F., Moreno M., Stadler M., Brönstrup M., Kirschning A., Müller R. (2017). Discovery and Total Synthesis of Natural Cystobactamid Derivatives with Superior Activity against Gram-Negative Pathogens. Angew. Chem. Int. Ed..

[10] Testolin G., Cirnski K., Rox K., Prochnow H., Fetz V., Grandclaudon C., Mollner T., Baiyoumy A., Ritter A., Leitner C. (2019). Synthetic studies of cystobactamids as antibiotics and bacterial imaging carriers lead to compounds with high in vivo efficacy. Chem Sci..

[11] Lukežič T., Lešnik U., Podgoršek A., Horvat J., Polak T., Šala M., Jenko B., Raspor P., Herron P.R., Hunter I.S. (2013). Identification of the chelocardin biosynthetic gene cluster from Amycolatopsis sulphurea: A platform for producing novel tetracycline antibiotics. Microbiology.

[12] Stepanek J.J., Lukežič T., Teichert I., Petković H., Bandow J.E. (2016). Dual mechanism of action of the atypical tetracycline chelocardin. Biochim. Biophys. Acta.

[13] Hennessen F., Miethke M., Zaburannyi N., Loose M., Lukežič T., Bernecker S., Hüttel S., Jansen R., Schmiedel J., Fritzenwanker M. (2020). Amidochelocardin Overcomes Resistance Mechanisms Exerted on Tetracyclines and Natural Chelocardin. Antibiotics.

[14] Colomb-Cotinat M., Lacoste J., Brun-Buisson C., Jarlier V., Coignard B., Vaux S. (2016). Estimating the morbidity and mortality associated with infections due to multidrug-resistant bacteria (MDRB), France, 2012. Antimicrob. Resist. Infect. Control..

[15] (2017). WHO Publishes List of Bacteria for Which New Antibiotics Are Urgently Needed. https://www.who.int/news/item/27-02-2017-who-publishes-list-of-bacteria-for-which-new-antibiotics-are-urgently-needed.

[16] Franke R., Overwin H., Häussler S., Brönstrup M., Traxler M.F. (2021). Targeting Bacterial Gyrase with Cystobactamid, Fluoroquinolone, and Aminocoumarin Antibiotics Induces Distinct Molecular Signatures in *Pseudomonas aeruginosa*. mSystems.

[17] Lukežič T., Fayad A.A., Bader C., Harmrolfs K., Bartuli J., Groß S., Lešnik U., Hennessen F., Herrmann J., Pikl Š. (2019). Engineering Atypical Tetracycline Formation in *Amycolatopsis sulphurea* for the Production of Modified Chelocardin Antibiotics. ACS Chem. Biol..

[18] Lešnik U., Lukežič T., Podgoršek A., Horvat J., Polak T., Šala M., Jenko B., Harmrolfs K., Ocampo-Sosa A., Martínez-Martínez L. (2015). Construction of a new class of tetracycline lead structures with potent antibacterial activity through biosynthetic engineering. Angew. Chem. (Int. Ed Engl.).

[19] Elgaher W.A.M., Hamed M.M., Baumann S., Herrmann J., Siebenbürger L., Krull J., Cirnski K., Kirschning A., Brönstrup M., Müller R. (2020). Cystobactamid 507: Concise Synthesis, Mode of Action, and Optimization toward More Potent Antibiotics. Chem. Eur. J..

[20] Michalczyk E., Hommernick K., Behroz I., Kulike M., Pakosz-Stępień Z., Mazurek L., Seidel M., Kunert M., Santos K., von Moeller H. (2023). Molecular mechanism of topoisomerase poisoning by the peptide antibiotic albicidin. Nat. Catal..

[21] Kleebauer L., Zborovsky L., Hommernick K., Seidel M., Weston J.B., Süssmuth R.D. (2021). Overcoming AlbD Protease Resistance and Improving Potency: Synthesis and Bioactivity of Antibacterial Albicidin Analogues with Amide Bond Isosteres. Org. Lett..

[22] Domenico P., Salo R.J., Cross A.S., Cunha B.A. (1994). Polysaccharide capsule-mediated resistance to opsonophagocytosis in Klebsiella pneumoniae. Infect. Immun..

[23] Ramos-Martín F., D’Amelio N. (2023). Drug resistance: An incessant fight against evolutionary strategies of survival. Microbiol. Res..

[24] Hobby C.R., Herndon J.L., Morrow C.A., Peters R.E., Symes S.J.K., Giles D.K. (2019). Exogenous fatty acids alter phospholipid composition, membrane permeability, capacity for biofilm formation, and antimicrobial peptide susceptibility in Klebsiella pneumoniae. Microbiologyopen.

[25] Ramos-Vivas J., Chapartegui-González I., Fernández-Martínez M., González-Rico C., Fortún J., Escudero R., Marco F., Linares L., Montejo M., Aranzamendi M. (2019). Biofilm formation by multidrug resistant Enterobacteriaceae strains isolated from solid organ transplant recipients. Sci. Rep..

[26] Thi M.T.T., Wibowo D., Rehm B.H. (2020). *Pseudomonas aeruginosa* Biofilms. Int. J. Mol. Sci..

[27] Whitfield C., Williams D.M., Kelly S.D. (2020). Lipopolysaccharide O-antigens-bacterial glycans made to measure. J. Biol. Chem..

[28] Post S.J., Shapiro J.A., Wuest W.M. (2019). Connecting iron acquisition and biofilm formation in the ESKAPE pathogens as a strategy for combatting antibiotic resistance. Medchemcomm.

[29] Benamara H., Rihouey C., Jouenne T., Alexandre S. (2011). Impact of the biofilm mode of growth on the inner membrane phospholipid composition and lipid domains in *Pseudomonas aeruginosa*. Biochim. Biophys. Acta.

[30] EUCAST (2022). European Committee on Antimicrobial Susceptibility Testing. https://www.eucast.org/clinical_breakpoints.

